# Multiple myeloma–derived miR‐27b‐3p facilitates tumour progression via promoting tumour cell proliferation and immunosuppressive microenvironment

**DOI:** 10.1002/ctm2.1140

**Published:** 2023-01-15

**Authors:** Xiaojing Wei, Zhen Yu, Peixia Tang, Hao Sun, Lixin Gong, Lanting Liu, Teng Fang, Yi He, Tingyu Wang, Weiwei Sui, Yan Xu, Gang An, Zhenshu Xu, Xiaoke Ma, Lugui Qiu, Mu Hao

**Affiliations:** ^1^ State Key Laboratory of Experimental Hematology, National Clinical Research Center for Blood Diseases, Haihe Laboratory of Cell Ecosystem, Institute of Hematology & Blood Diseases Hospital Chinese Academy of Medical Sciences & Peking Union Medical College Tianjin China; ^2^ Tianjin Institutes of Health Science Tianjin China; ^3^ School of Computer Science and Technology Xidian University Xi'an China; ^4^ Department of Hematology The Second Hospital of Tianjin Medical University Tianjin China; ^5^ Hematology Department Fujian Medical University Union Hospital Fujian Institute of Hematology Fuzhou China

Dear Editor

Multiple myeloma (MM) is still an incurable plasma cells malignancy. Immunotherapy has proven to be very encouraging in the therapy of cancers especially in haematological malignancy, including MM.^1^ However, the efficacy of immunotherapy on MM remains unsatisfactory. Tumour‐promoting inflammation and avoiding immune destruction are hallmarks of cancer. The immunosuppressive tumour microenvironment (iTME) is critical for the survival, proliferation and anti‐immunotherapy of MM.^2^ However, the molecular mechanism underlying the formation of iTME in MM and the interaction between tumour and immune cells has not been fully understood. Here, we identified the aberrant level of miR‐27b‐3p was a liquid biomarker for the progression of MM and clarified miR‐27b‐3p role in MM progression either targeted CD28 on T cells and FBXW7 on tumour cells. We also highlighted that miR‐27b‐3p were encapsulated in exosome and played pivotal roles in the interaction between tumour and immune cells, which is critical for the formation of iTME in MM. Our results demonstrated that miR‐27b‐3p plays pivotal roles in myelomagenesis and would be an ideal therapeutic target in myeloma.

In this study, we confirmed that the population of immunosuppressive T cells was significantly increased in the MM microenvironment by using multicolour flow cytometry to examine the T‐cell proportion in the peripheral blood (PB) and bone marrow (BM) of newly diagnosed MM (NDMM) patients and healthy donors (HDs) (Figure 1A–H, Tables S1 and S2). Consistently,^3^, ^4^ our study confirmed that T‐cell differentiation and function were deregulated by MM cell infiltration (Figure 1I–K).

**FIGURE 1:**
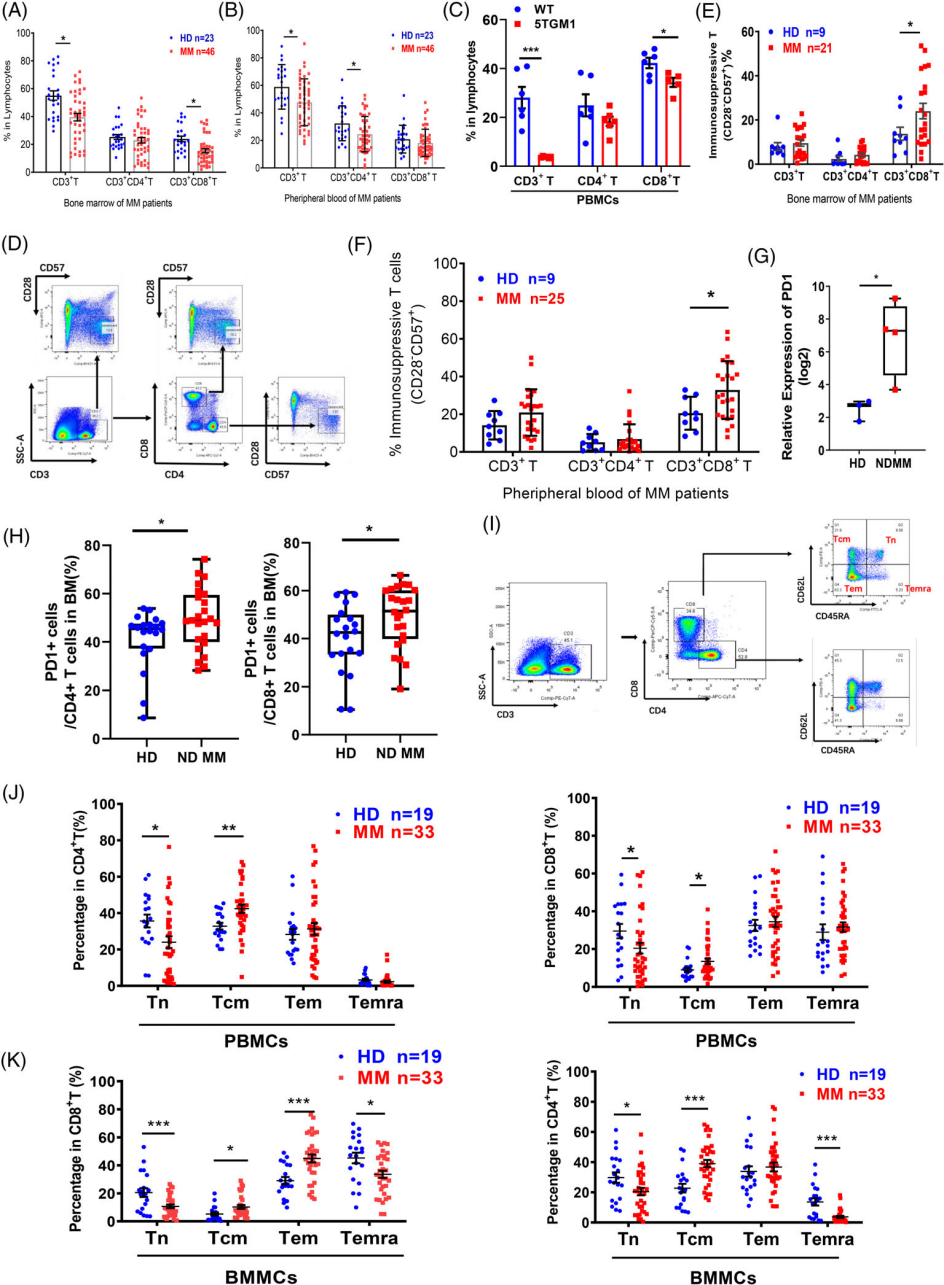
Immunosuppressive T cells significantly increased in microenvironment of multiple myeloma (MM). (A and B) Flow cytometry analysis of the proportion of CD3^+^ T cell subtypes in BMMCs (A) and PBMCs (B) from MM patients (*n* = 46) compared with healthy donors (HDs) (*n* = 23) (*p* < .001, *t* test). (C) Bar charts represent the mean percentage of PBMCs’ T cell from 5TGM1 (*n* = 6) compared with wide type (WT) (*n* = 6) (*p < .05, **p < .01, ***p < .001, *t* test). (D) Flow cytometry was used to detect the immunosuppressive T cells (CD28^−^CD57^+^) in CD3^+^ T subsets of BMMCs and PBMCs from MM patients and HDs. (E and F) Bar charts represent the mean proportions of the immunosuppressive T cells (CD28^−^CD57^+^) in CD3^+^ T subsets of BMMCs (E) and PBMCs (F) from MM patients and HDs (MM *n* = 21 or 25; HD *n* = 9) (*p* < .05, *t* test). (G) Bar charts represent the mean expression level of PD1 in CD3^+^ T cells of newly diagnosed MM (NDMM) and HD's bone marrow. (H) Flow cytometry analysis of the proportion of PD1^+^ cell in CD4^+^ T cell and CD8^+^ T cell of NDMM and HD's bone marrow (*p* < .05, *t* test). (I) Flow cytometry was used to detect the Tn, Tcm, Tem and Temra in CD3^+^CD8^+^ T or CD3^+^CD4^+^ T cell subtypes of BMMCs and PBMCs (Tn: naïve T cell; Tcm: central memory T cell; Tem: effector memory T cell and Temra: effector T cell). (J) Bar charts represent the mean percentage of Tn, Tcm, Tem and Temra in CD3^+^CD8^+^ T or CD3^+^CD4^+^ T cell subtypes of PBMCs from MM patients (*n* = 33) compared with HDs (*n* = 19) (*p < .05, **p < .01, ***p < .001, *t* test). (K) Bar charts represent the mean percentage of Tn, Tcm, Tem and Temra in CD3^+^CD8^+^ T or CD3^+^CD4^+^ T cell subtypes of BMMCs from MM patients (*n* = 33) compared with HDs (*n* = 19) (*p < .05, **p < .01, ***p < .001, *t* test).

As previously reported,^5^, ^6^, ^7^ miRNAs have been implicated in intercellular crosstalk within the iTME in many malignancies. MiRNAs encapsulated in exosomes downregulate T‐cell responses through decreased T‐cell receptor signalling and diminished cytokine and granzyme B secretions. However, the study of the roles of exosome and encapsulated miRNAs in MM still are limited. More recent studies suggest that circulating cell‐free microRNAs and circulating exosomes represent the major components of liquid biopsy assays in clinical practice. Here, our study intent to explore the crosstalk between MM cells and the microenvironment, especially between the tumour and immune cells. Hence, we utilized the tumour cells isolated from BM samples by CD138 MicroBeads (Miltenyi Biotec, Germany) along with serum samples, and the pairwise miRNA profiling was performed via small RNA‐seq. Our data indicated that the levels of serum circulatory miRNAs (scmiR‐27b‐3p, scmiR‐145‐3p, scmiR‐628‐3p, scmiR‐342‐5p and scmiR‐30e‐3p) were significantly decreased in NDMM patients (Figure S1). Kaplan–Meier survival analysis showed that the MM patients with decreased levels of scmiR‐27b‐3p, scmiR‐145‐3p and scmiR‐628‐3p (Figure S2a,b) had a dismal outcome compared with the other ones. The abnormal expression pattern of miRNA can be used as a tool to stratify the prognosis of MM (Figure S2c,d).

Next, the results indicated that a lower level of scmiR‐27b‐3p corresponds with a lower proportion of CD3^+^/CD3^+^CD4^+^ T cells and a higher proportion of immunosuppressive CD3^+^/CD3^+^CD8^+^ T cells (CD3^+^/CD3^+^CD8^+^CD28^−^CD57^+^) in MM patients (Figure 2A–F). Functional analyses showed that CD3^+^CD8^+^ T cells in MM patients could not be effectively activated by human CD3/CD28 T cell activator (STEMCELL, Figure 2G). These findings indicate that the decreased level of serum circulatory miR‐27b‐3p could be used as an effective biomarker of T‐cell dysfunction that is probably related to the iTME in MM.

**FIGURE 2:**
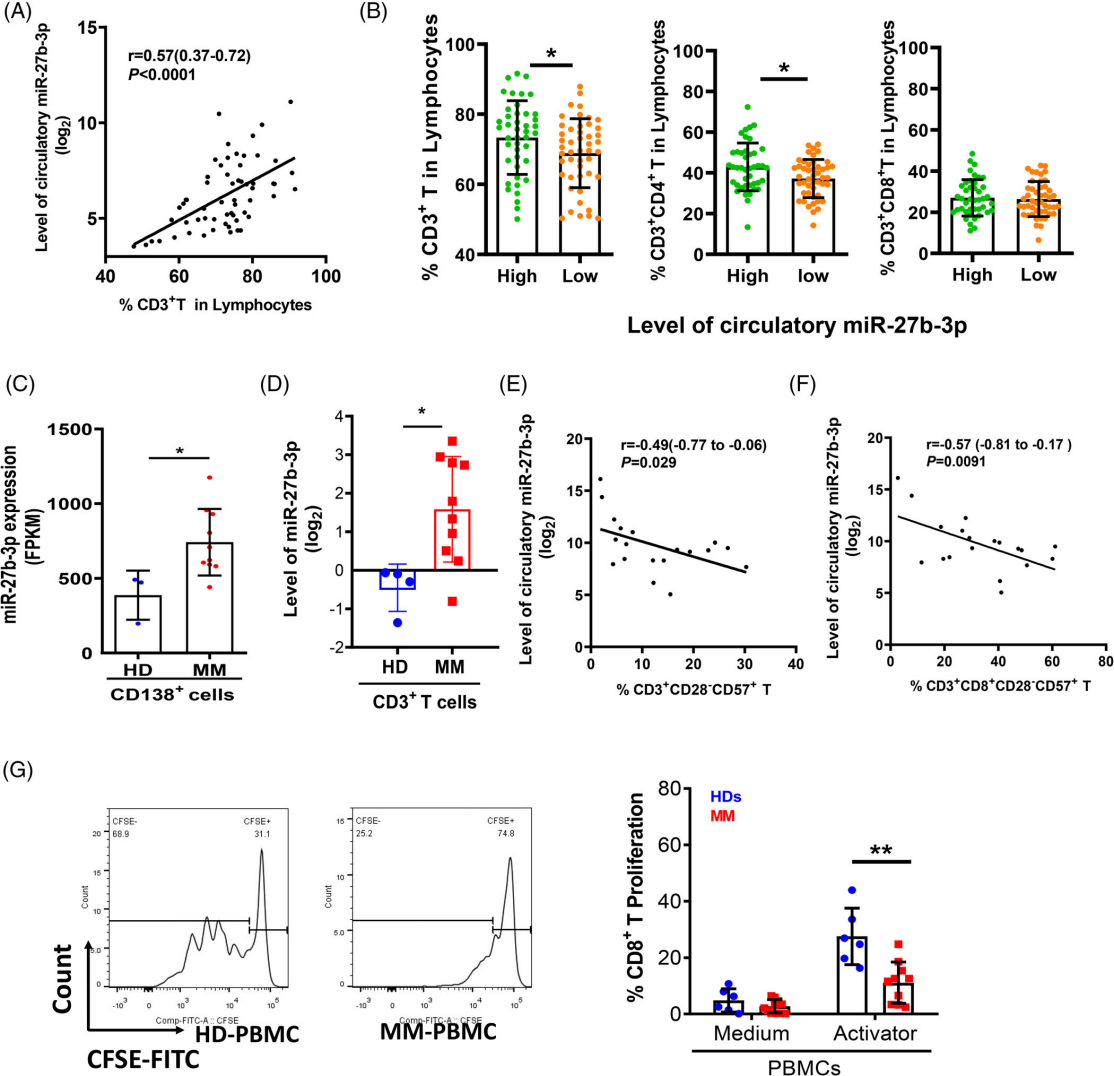
Decreased level of serum circulatory miR‐27b‐3p highly correlated with poor survival and T cell immunosuppression in multiple myeloma (MM): (A) Spearman correlation analysis between the level of serum circulatory miR‐27b‐3p and the proportion of CD3^+^ T cells in peripheral blood (PB) lymphocytes (*n* = 60) (*r* = .57, *p* < .0001); (B) the proportion of CD3^+^ T cell subtypes in PB lymphocytes of MM patients was associated with the levels of circulatory miR‐27b‐3p (high group *n* = 43; low group *n* = 47); (C) level of miR‐27b‐3p in CD138^+^ primary MM cells compared to normal plasma cells by RNA‐seq (HD *n* = 3; MM *n* = 10) (*p* < .05, *t* test); (D) the level of miR‐27b‐3p was examined by RT‐qPCR in CD3^+^ T cells from MM patients (*n* = 10) and healthy donors (HDs) (*n* = 4) (*p* < .05, *t* test); (E and F) the immunosuppressive phenotype (CD28^−^CD57^+^) of CD3^+^ T cells (*r* = −.49, *p* = .029) (*n* = 20) (G) and CD3^+^CD8^+^ T cells (*r* = −.57, *p* = .0091) (*n* = 20) (H) was negatively correlated with the levels of serum circulatory miR‐27b‐3p; (G) CFSE staining and flow cytometry analysis of the proliferation ability of CD8^+^ T cells. Compared with HD T cells (*n* = 6), the activation and proliferation of CD3^+^CD8^+^ T cells in PBMCs of newly diagnosed MM (NDMM) patients (*n* = 10) were decreased after CD3/CD28 T cell activator treatment (*p* < .05, *t* test). Error bars represent the mean ± SD of three independent experiments (*p < .05, **p < .01).

MiRNAs encapsulated in exosomes contribute to the crosstalk between tumour cells and the microenvironment.^8^ The exosomes were isolated^9^ from MM/HD serum (Figure S3) and the culture supernatant of MM cell lines by exoRNeasy serum/plasma midi kits (QIAGEN). As the guideline by MISEV2018 and IVES mentioned,^9^ three positive protein markers (Tsg101, CD63 and CD9) and two negative protein markers (GOLGA2 and Calnexin) were detected by western blots to confirm the exosome isolation and determine the distribution of miR‐27b‐3p (Figure S4). The density of the exosome was 8.13e^10^ ± 2.16e^10^/ml. These results clearly showed that miR‐27b‐3p was efficiently encapsulated in exosomes and released into circulation. Confocal microscopy analysis showed that the exosomes were transferred to CD3^+^ T cells in the co‐culture system of PKH67‐labelled exosomes and primary CD3^+^ T cells from the PB of HDs (Figure S5a). The proportion of CD28^−^CD57^+^ immunosuppressive T cells was significantly increased in CD3^+^ T cells after 7‐day co‐culture with exosomes, which isolated from MM patient serum, and miR‐27b‐3p‐OE MM cells (miRNA mimic, Guangzhou RiboBio Co., Ltd.), respectively. The results were consistent with the increasing miR‐27b‐3p level in T cells (Figure S5b–g). Altogether, our findings demonstrate that miR‐27b‐3p encapsulated in exosomes could be taken up by surrounding CD3^+^ T cells. The increasing miR‐27b‐3p causes T‐cell dysfunction.

To clarify the mechanism by which miR‐27b‐3p regulates the immunosuppressive phenotype of T cells, we focused on the CD28 and FBXW7 genes that predicted by TargetScan, and so on (Figure S6a), and confirmed by luciferase reporter assays (Figures 3A and S6b). The expression of CD28 (the prominent costimulatory molecule on T cells) was significantly decreased at both the mRNA and protein level after miR‐27b‐3p upregulation in Jurkat T cell line or primary CD3^+^ T cells from HD PBMCs (Figure 3B–D). The function of T cells, as determined by the production of cytokines (IL‐2 and IFN‐γ), was significantly suppressed, accompanied by decreased proliferation, in the miR‐27b‐3p OE T cells compared with the empty vector control (Figure 3E–H). Additionally, blocking exosome release in miR‐27b‐3p OE MM cell (RPMI8226) by GW4869 treatment in the co‐culture system can significantly prevent the immunosuppression of T cells as well. The proliferation and cytokine secretion (IL‐2 and IFN‐γ) in CD3^+^ T cell were recovered (Figure 3I–K). Of note, inhibit miR‐27b‐3p expression (miRNA inhibitor, Guangzhou RiboBio Co., Ltd.) in MM cell (RPMI8226), the proportion of CD3^+^CD28^−^CD57^+^ immunosuppressive T cells was significantly decreased after the co‐culture, the proliferation and cytokines production of T cells were recovered (Figure 3L–N). These findings supported that miR‐27b‐3p encapsulated in exosome play pivotal roles in the crosstalk between MM and T cells. MiR‐27b‐3p promotes the immunosuppressive dysfunction of T cells by downregulating the expression of CD28.

**FIGURE 3:**
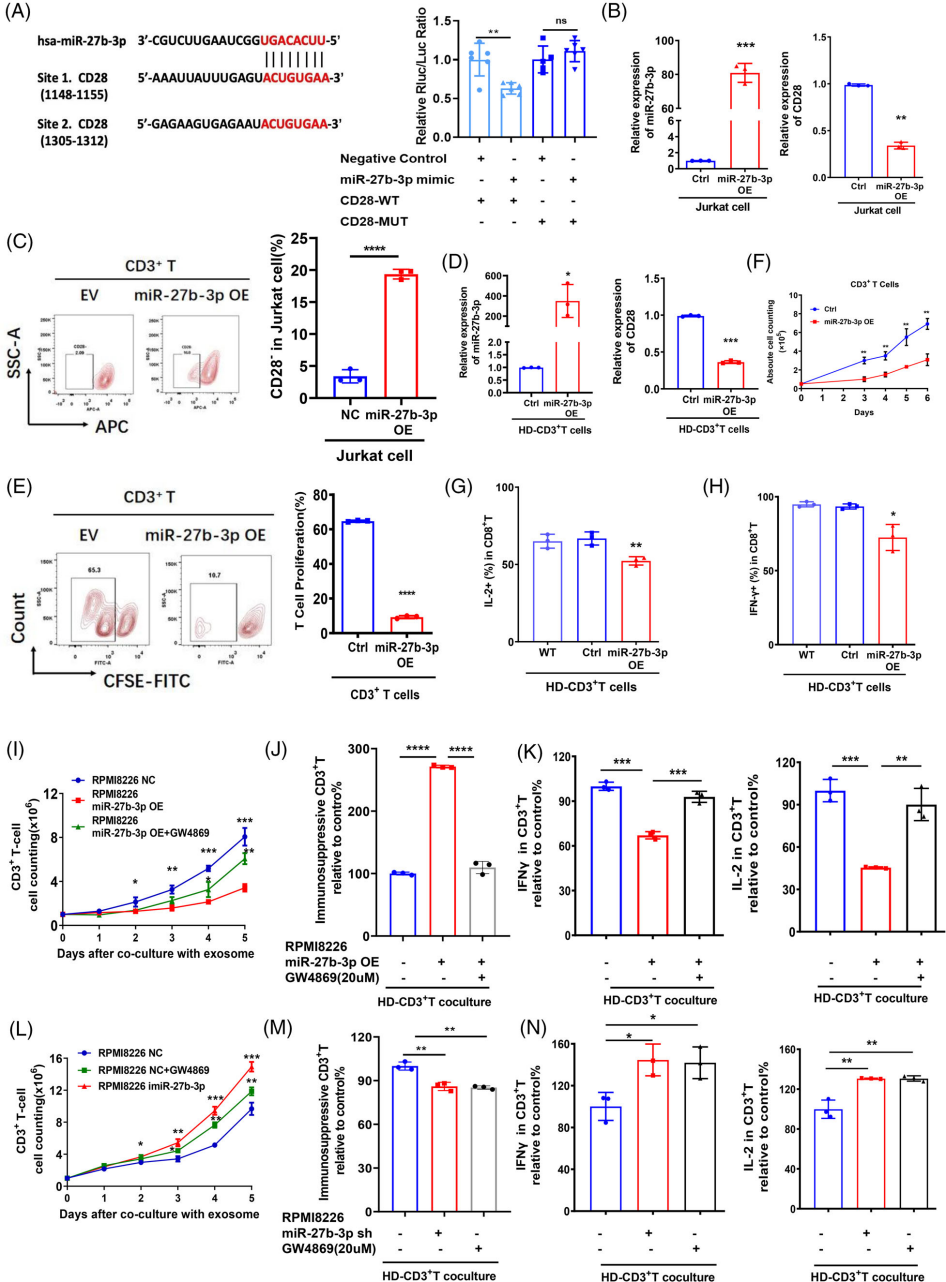
MiR‐27b‐3p promotes T cell dysfunction by targeting CD28 expression: (A) Two binding sites analysis of miR‐27b‐3p with CD28 mRNA based on the TargetScan database analysis. Dual luciferase assay was performed in 293T cells to confirm that CD28 was the target gene of miR‐27b‐3p in T cells. (B) The levels of miR‐27b‐3p and CD28 were detected by RT‐qPCR in the miR‐27b‐3p‐OE Jurkat cell line. (C) Flow cytometry analysis shows the percentage of CD28^−^ in miR‐27b‐3p‐OE Jurkat T cell line compared with empty vector (EV) control. (D) After overexpressing miR‐27b‐3p in healthy donor (HD) primary CD3^+^ T cells, the levels of miR‐27b‐3p and CD28 were detected by RT‐qPCR. (E) The proliferation of T cells was examined in miR‐27b‐3p OE primary CD3^+^ T cells activated by a CD3/CD28 activator. (F) The cell growth of CD3^+^ T cells with miR‐27b‐3p OE was detected by trypan blue exclusion assay. (G and H) The levels of the cytotoxic cytokines IL‐2 and IFN‐γ were detected by flow cytometry analysis in CD3^+^CD8^+^ T cells with miR‐27b‐3p overexpression after activation by CD3/CD28 activator. NS, no significance. Error bars represent the mean ± SD of three independent experiments (*p < .05, **p < .01, ***p < .001, *t* test). (I–K) After blocking exosome release treated with GW4869 (20 μM) in miR‐27b‐3p OE RPMI 8226 cells, the exosome was isolated and co‐cultured with CD3^+^ T cell from HD for 72 h. The function of T cell was examined, including the proliferation of T cell (I), the percentage of immunosuppressive cells (CD28^−^CD57^+^) in CD3^+^ T cell (J) and the ability to secrete cytokines (IL‐2, IFN‐γ) of T cell (K). (L–N) After blocking exosome release treated with GW4869 (20 μM) or inhibiting miR‐27b‐3p (imiR‐27b‐3p) expression transfected with complementary single strand of miR‐27b‐3p in RPMI 8226 cells, the exosome was isolated and co‐cultured with CD3^+^ T cell from HD for 72 h. The function of T cell was examined, including the proliferation of T cell (L), the percentage of immunosuppressive cells (CD28^−^CD57^+^) in CD3^+^ T cell (M) and the ability to secrete cytokines of T cell (IL‐2, IFN‐γ) (N).

FBXW7 is an E3 ubiquitin ligase that regulates the ubiquitin‐mediated degradation of oncoproteins such as MYC and confers tumorigenicity in MM.^10^ Our data showed that miR‐27b‐3p OE promoted MM cell growth by targeting FBXW7 mRNA expression. Level of MYC was notably increased (Figure 4A–C). Moreover, Kaplan–Meier analysis showed that MM patients with a low level of FBXW7 had dismal outcome compared with high ones (Figure 4D) in the GEO dataset (GSE2658) and MMRF‐CoMMpass database. Therefore, our study confirmed that miR‐27b‐3p promotes MM cell proliferation by downregulating FBXW7, which promoted the stabilization and accumulation of MYC in MM cells. Furthermore, in 5TGM1 murine myeloma mode, all of the mice in miR‐27b‐3p OE group were dead within 42 days; however, there was only one mouse dead in control group at the same time. The mean survivability between miR‐27b‐3p OE group and control group was 46.2 versus 38.6 days (*p* = .018, Figure 4K). Therefore, our data demonstrated that miR‐27b‐3p overexpression promoted MM cell growth, which caused the increase of tumour burden and the shorter survival of tumour‐bared mice.

**FIGURE 4:**
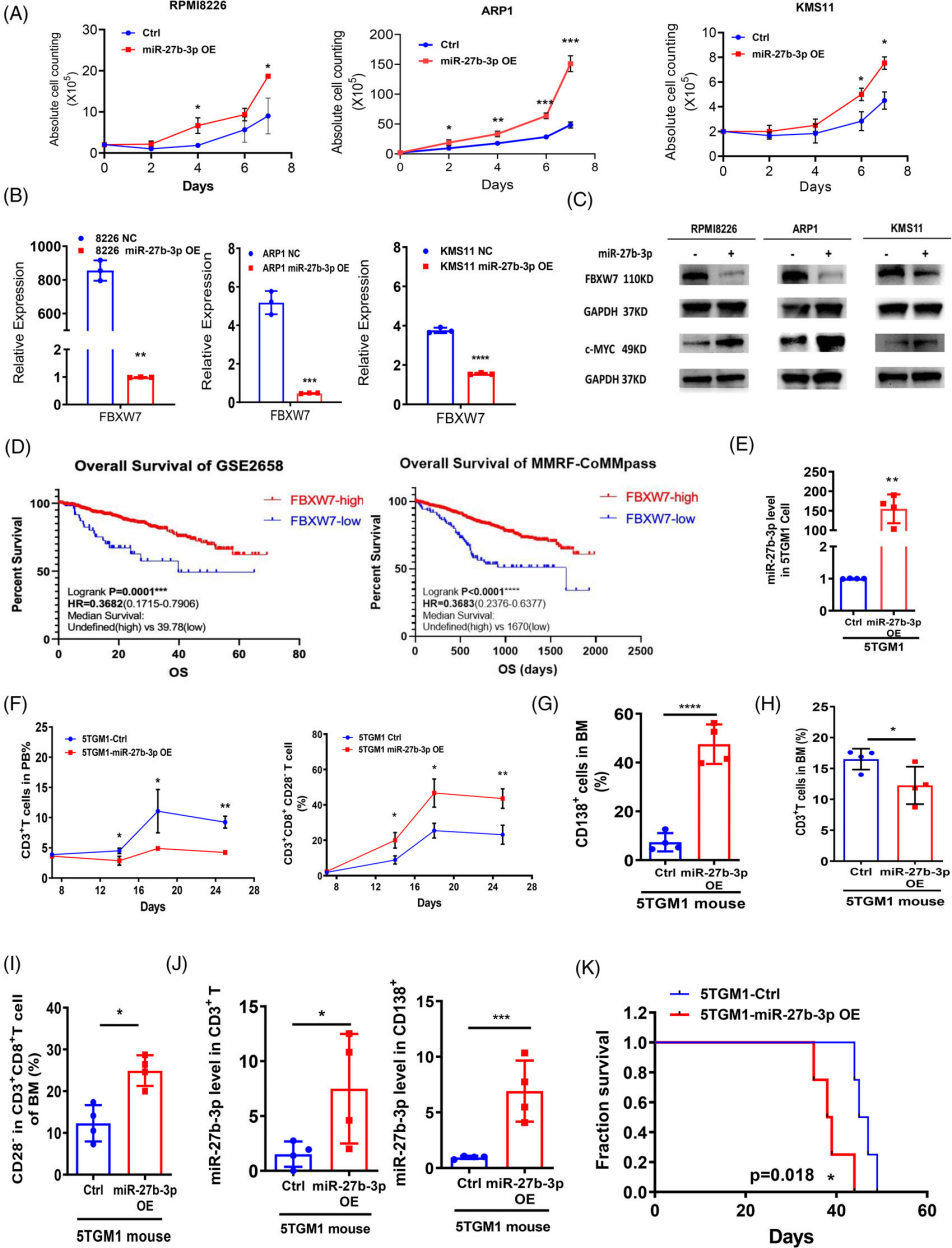
Overexpression of miR‐27b‐3p promotes multiple myeloma (MM) cell proliferation via downregulating tumour suppressor gene FBXW7: (A) The cell proliferation of miR‐27b‐3p‐OE MM cell lines (RPMI8226, ARP1, KMS11) was measured by absolute cell counts; (B) FBXW7 expression level was detected by RT‐qPCR in miR‐27b‐3p‐OE MM cells and control cells. Error bars represent the mean ± SD of three independent experiments (*p < .05, **p < .01, ***p < .001); (C) Western blots were conducted in MM cells to detect FBXW7 and c‐MYC protein level; (D) Kaplan–Meier analysis of MM patient survival based on FBXW7 levels in GSE2658 and MMRF‐CoMMpass databases (logrank test, *p* < .001); (E) In vivo study performed in the 5TGM1 murine model. MiR‐27b‐3p OE 5TGM1 MM cells were constructed; (F) After MiR‐27b‐3p OE 5TGM1 cell transplantation, CD3^+^ T cells and immunosuppressive T cells (CD3^+^CD8^+^CD28^−^) in peripheral blood (PB) were monitored by flow cytometry. (G–I) CD138^+^ MM cells (G), CD3^+^ T cells (H) and CD28^−^ T cells (I) from mice bone marrow (BM) were monitored by flow cytometry after 5TGM1 cell transplantation; (J) the levels of miR‐27b‐3p were detected in CD138^+^ MM cells and CD3^+^ T cells of mice BM by RT‐qPCR. Error bars represent the mean ± SD of three independent experiments (*p < .05, **p < .01, ***p < .001); (K) Kaplan–Meier analysis of the survival of tumour‐bearing mice. Mean survivability survival rate between the control and miR‐27b‐3p OE mice was analysed (logrank test, *p* < .05).

In summary, our findings demonstrate that serum circulatory miR‐27b‐3p acts as a useful biomarker for MM prognosis evaluation as well as clarifies the function of miR‐27b‐3p function in myeloma immunosuppressive status. Our results support the conclusion that miR‐27b‐3p is a potential therapeutic target in MM, and MM cell crosstalk with immune cells via exosomes is an important mechanism in the formation of the iTME. This study is beneficial for better understanding miRNA trafficking and its roles in MM pathogenesis, particularly in myeloma cells driving immune escape. The working model of this study is shown in Figure S7.

## CONFLICTS OF INTEREST

The authors report no conflicts of interest.

## Supporting information

Supporting Information

Figures S1–S7
