# Supplementary material for: Multiple myeloma–derived miR‐27b‐3p facilitates tumour progression via promoting tumour cell proliferation and immunosuppressive microenvironment
Source: Clin Transl Med. 2023 Jan 15;13(1):e1140. doi: 10.1002/ctm2.1140 (PMC9841122; doi:10.1002/ctm2.1140)
Supplement: Supplementary file 1 — Supporting Information [file CTM2-13-e1140-s001.docx]

**Supplemental Fig.1 MiRNAs profiles in primary MM cells and serum of MM patients.**

(a) PCA analysis of CD138^+^ primary cells sample. Red represents CD138^+^ primary MM cells.

(b) Heatmap of the miRNA profiles of CD138^+^ primary MM cells in NDMM patients (dark yellow, n=8) and the corresponding control cells in HDs (light yellow, n=2). Red represents upregulated miRNAs, and blue represents downregulated miRNAs.

(c) PCA analysis of serum sample. Red represents serum sample of MM patients.

(d) Heatmap of circulatory miRNA profiles of serum samples from NDMM patients (dark yellow, n=8) and HDs (light yellow, n=2). Red represents upregulated miRNAs, and blue represents downregulated miRNAs.

(e) Venn diagram shows the 36 common DEmiRs in both CD138^+^ cells and serum samples (NDMM patients vs. HDs).

(f) Heatmap shows the variation pattern of 36 miRNAs in all primary MM cells and serum samples. The color on the top represents the kind of sample. Light red represents the HD serum. Dark red represents NDMM patient serum. Light yellow represents CD138^+^ cells from HDs. Dark yellow represents CD138^+^ cells from NDMM patients.

(g) The signaling pathways involved in the 36 DEmiRs were assessed by DIANA-miRPath analysis.

(h) Differential expression of circulatory miRNAs was validated by RT-qPCR in a large cohort of NDMM patients (n=201) and HDs (n=60). A significant decrease was observed in miR-27b-3p, miR-145-3p, miR-628-3p, miR-342-5p and miR-30e-3p in NDMM patient serum. Error bars represent the mean ± SD (*, P<0.05; **, P<0.01; ***, P<0.001, *t* test).

**Supplemental Fig.2 Prognostic value of serum circulatory miRNAs in patient with MM.**

(a&b) Prognostic value of circulatory miRNAs in MM patients. Progression-free survival (PFS)(a) and overall survival (OS)(b) were determined for MM patients according to the expression of circulatory miR-27b-3p, miR-145-3p, miR-628-3p. Survival analysis was determined via Kaplan–Meier analysis, and differences between curves were analyzed by the log-rank test. The significance threshold was defined as P < 0.05 (log rank).

(c) Patients with any two miRNAs (miR-27b-3p/miR-145-3p/miR-628-3p) low expression had worse survival including PFS and OS. PFS and OS were determined for NDMM patients according to the number of miRNAs low expression. Survival analysis was determined via Kaplan–Meier analysis, and differences between curves were analyzed by the log-rank test. The significance threshold was defined as P < 0.05 (log rank).

(d) Any two miRNAs (miR-27b-3p/miR-145-3p/miR-628-3p) low expression further predictive prognostic stratification for MM patients with R-ISS II. PFS and OS were determined for NDMM with R-ISS II patients according to the number of miRNAs low expression. Survival analysis was determined via Kaplan–Meier analysis, and differences between curves were analyzed by the log-rank test. The significance threshold was defined as P < 0.05 (log rank).

**Supplemental Fig.3 Serum exosome isolation and confirmation**

(a) Representative Transmission electron microscopy image of exosome bodies isolated from the serum of MM patients (scale bar=200 nm).

(b) The size of exosomes was detected by NanoSight dynamic light scattering analysis.

(c) Western blot was used to confirm marker protein expression on exosomes.

**Supplemental Fig.4 MiR-27b-3p efficiently encapsulates in exosomes and released to circulation.**

(a) RT-qPCR detection of the relative expression of miR-27b-3p in circulatory (Cir) MM patient serum and exosomes (Exo)(n=10) (P<0.05, t test).

(b) RT-qPCR detection of miR-27b-3p levels in serum exosomes from MM patients (n=11) and HDs (n=9) (P<0.05, *t* test).

(c&d) The miR-27b-3p-overexpressed (OE) MM cell lines (RPMI8226、KMS11、H929、MM1S and ARP1) were constructed (P<0.05, *t* test). The level of miR-27b-3p in exosomes of MM cell lines was detected by RT-qPCR (P<0.05, t test).

(e&f) After blocking exosome release by GW4869, RT-qPCR was performed to detect the level of miR-27b-3p in MM cells(e) and exosomes(f) (P<0.05, t test). Error bars represent the mean± SD of three independent experiments (*, P<0.05; **, P<0.01).

(g) There were two binding sites of miR-27b-3p with FBXW7 mRNA based on the database analysis (TargetScan). Dual luciferase assay was performed in 293T cells to confirm that FBXW7 was the target gene of miR-27b-3p in MM cells.

**Supplemental Fig.5 MiR-27b-3p transfers from exosome to T cells and facilitates T cell immunosuppression.**

(a) Immunofluorescence staining showed that exosomes were taken up by T cells after coculture. Green represents exosomes stained with PKH67. Red represents β-actin. Yellow is the merged image. CD3^+^ T cells were stained by using DAPI (nuclei) and β-actin. (Scale bars, 10 µm)

(b) CD3^+^ T cells were isolated from HD peripheral blood by MACS and cocultured with serum exosomes derived from MM patients for 7 days. The level of miR-27b-3p in CD3^+^ T cells after coculture with serum exosomes from MM patients detected by RT-qPCR (n=5) (P<0.05, t test).

(c) The immunosuppressive phenotype (CD28^-^CD57^+^) of T cells (CD3^+^ T and CD3^+^CD8^+^ T subtypes) was detected by flow cytometry after coculture for 7 days (n=5) (P<0.05, t test).

(d) PBMCs from HD were cocultured with serum exosomes from MM patients. CD3^+^ T and CD3+CD8+ T cells were detected by flow cytometry after 7 days of coculture (n=5) (P<0.05, t test).

(e) The immunosuppressive CD3^+^CD8^+^ T cells (CD3^+^CD8^+^CD28^-^CD57^+^) were detected in PBMCs after coculture with serum exosomes from MM patients, (n=5) (P<0.05, t test).

(f) MiR-27b-3p OE MM(ARP1) cell-derived exosomes were cocultured with PBMCs from healthy donors. Flow cytometry detection of CD3^+^ T and immunosuppressive T cells.

(g) MiR-27b-3p level was detected in PBMCs after coculture with exosomes from miR-27b-3p-OE MM(ARP1) cell line. Error bars represent the mean ± SD of three independent experiments (P<0.05, t test) (*, P<0.05; **, P<0.01; ***, P<0.001; ****, P<0.0001).

**Supplemental Fig.6 The bioinformatics analysis for the mRNA targets of miR-27b-3p.**

Venn diagram showed that 15 potential mRNA targets of miR-27b-3p that were predicted through the five independent database analysis (TargetScan, miRDB, microT- CDS, Tarbase, sequence database of our group).

**Supplemental Fig.7 The working model of this study.**

**Suppl. Table 1 The base-line characteristics of 209 NDMM patients.**

ISS: International Staging system

R-ISS: Revised International Staging system

**Suppl. Table 2 The base-line characteristics of 60** **healthy donor.**

|  | **HD** |
| --- | --- |
| **Number** | **60** |
| **Gender: males-females** | **53.3%-46.7%** |
| **Age median(min-max)**  **「years」** | **40.5(20-51)** |
