# Supplementary figures and images for: Multiple myeloma–derived miR‐27b‐3p facilitates tumour progression via promoting tumour cell proliferation and immunosuppressive microenvironment
Source: Clin Transl Med. 2023 Jan 15;13(1):e1140. doi: 10.1002/ctm2.1140 (PMC9841122; doi:10.1002/ctm2.1140)

## Slide 1
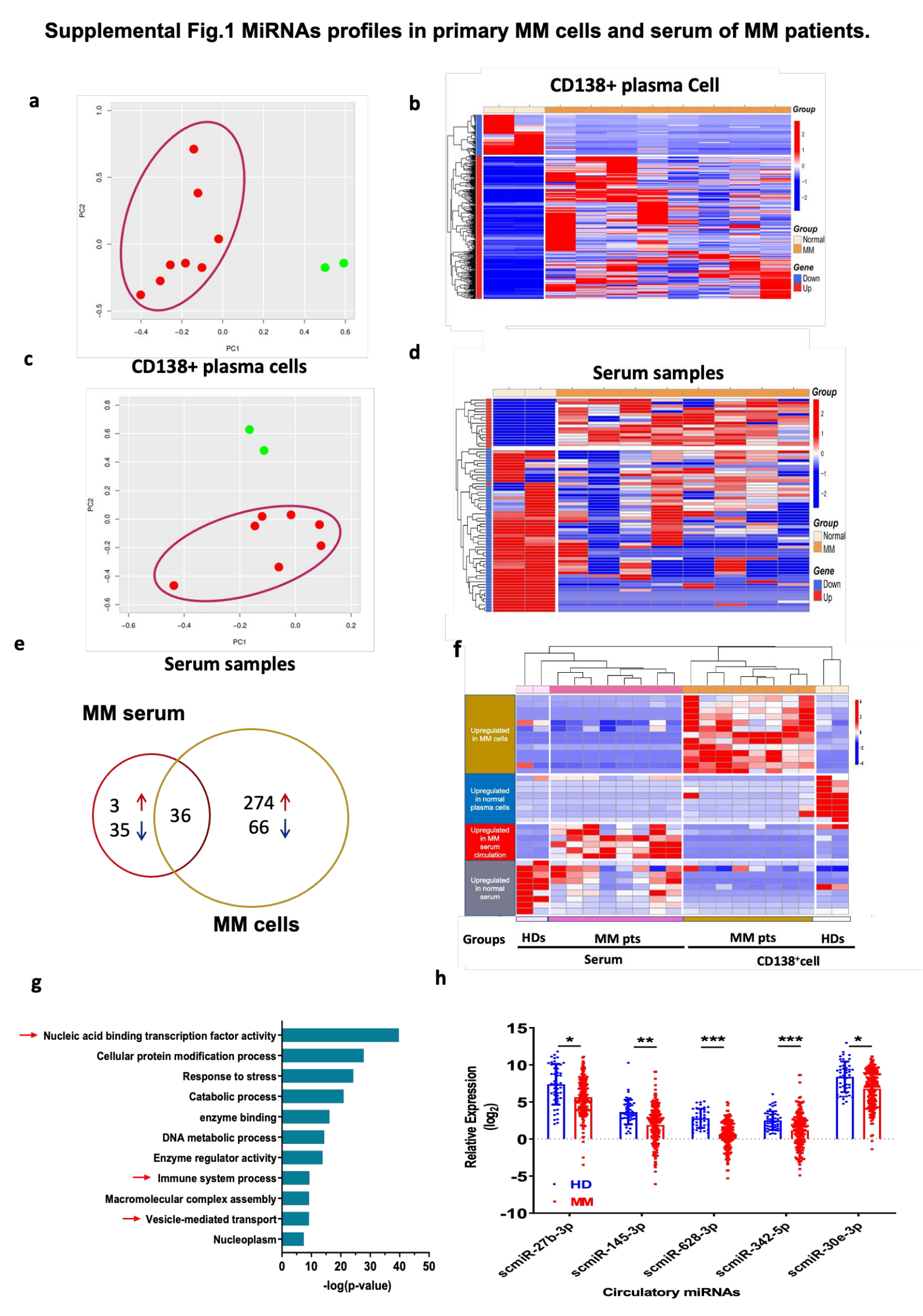

## Slide 2
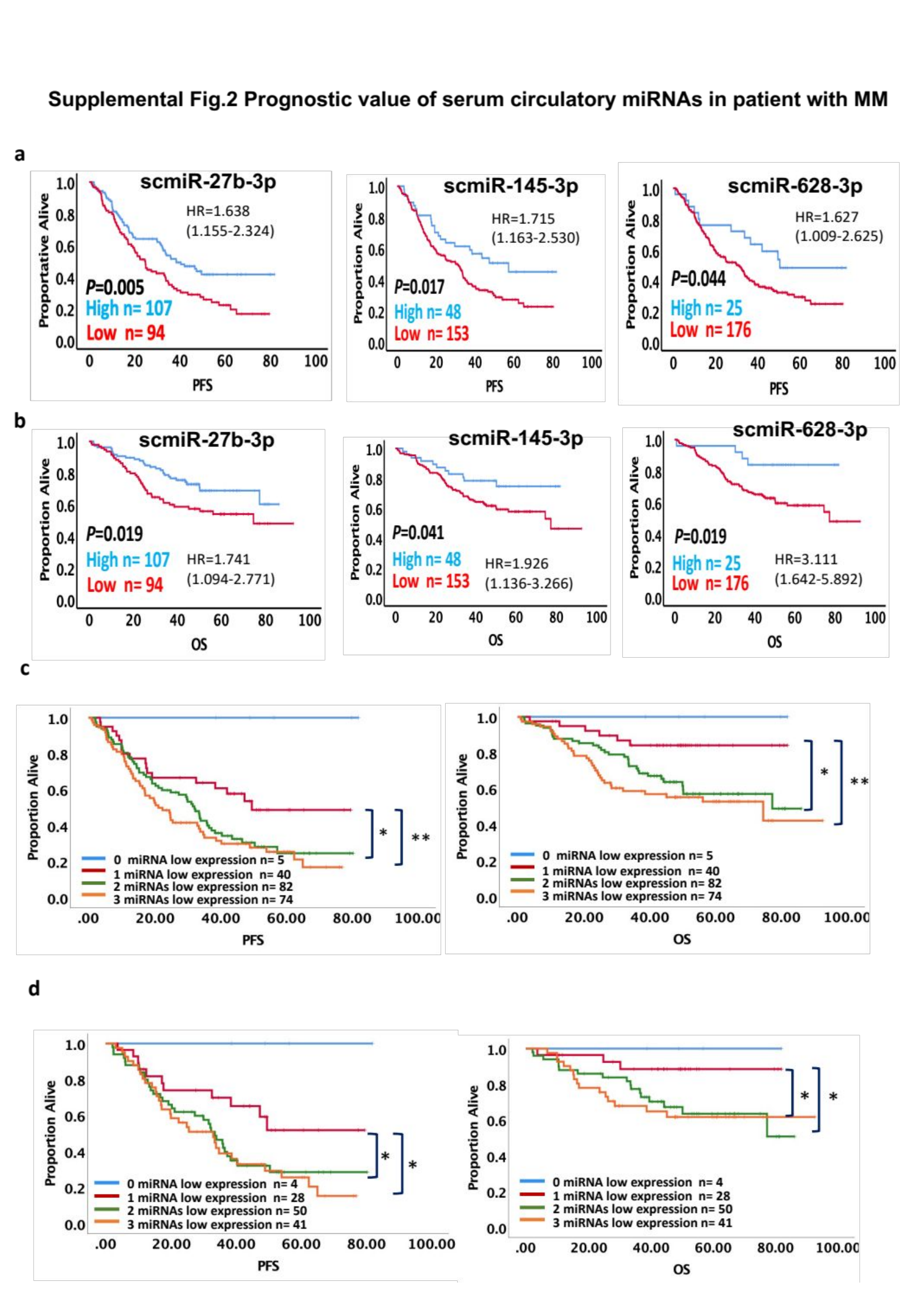

## Slide 3
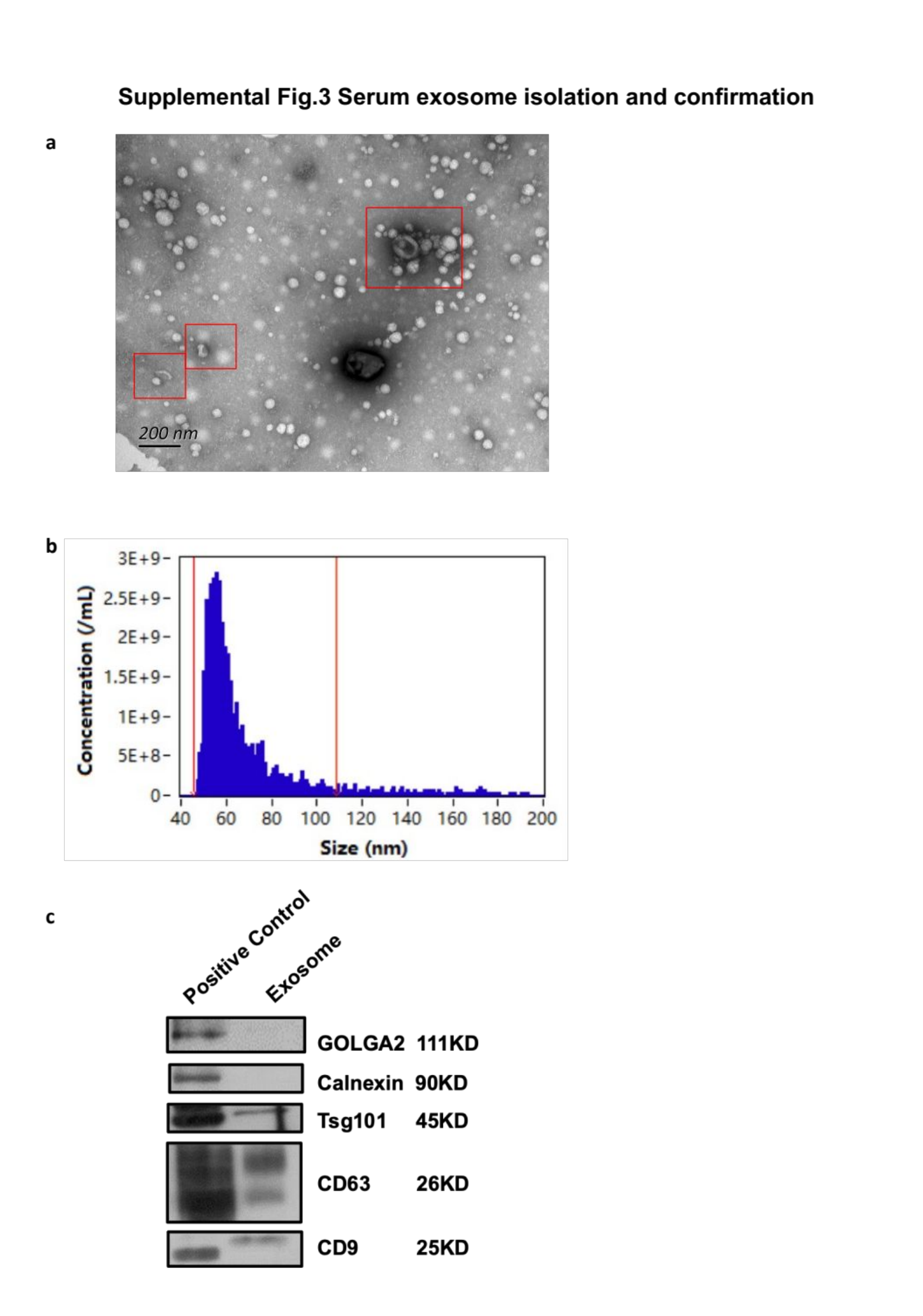

## Slide 4
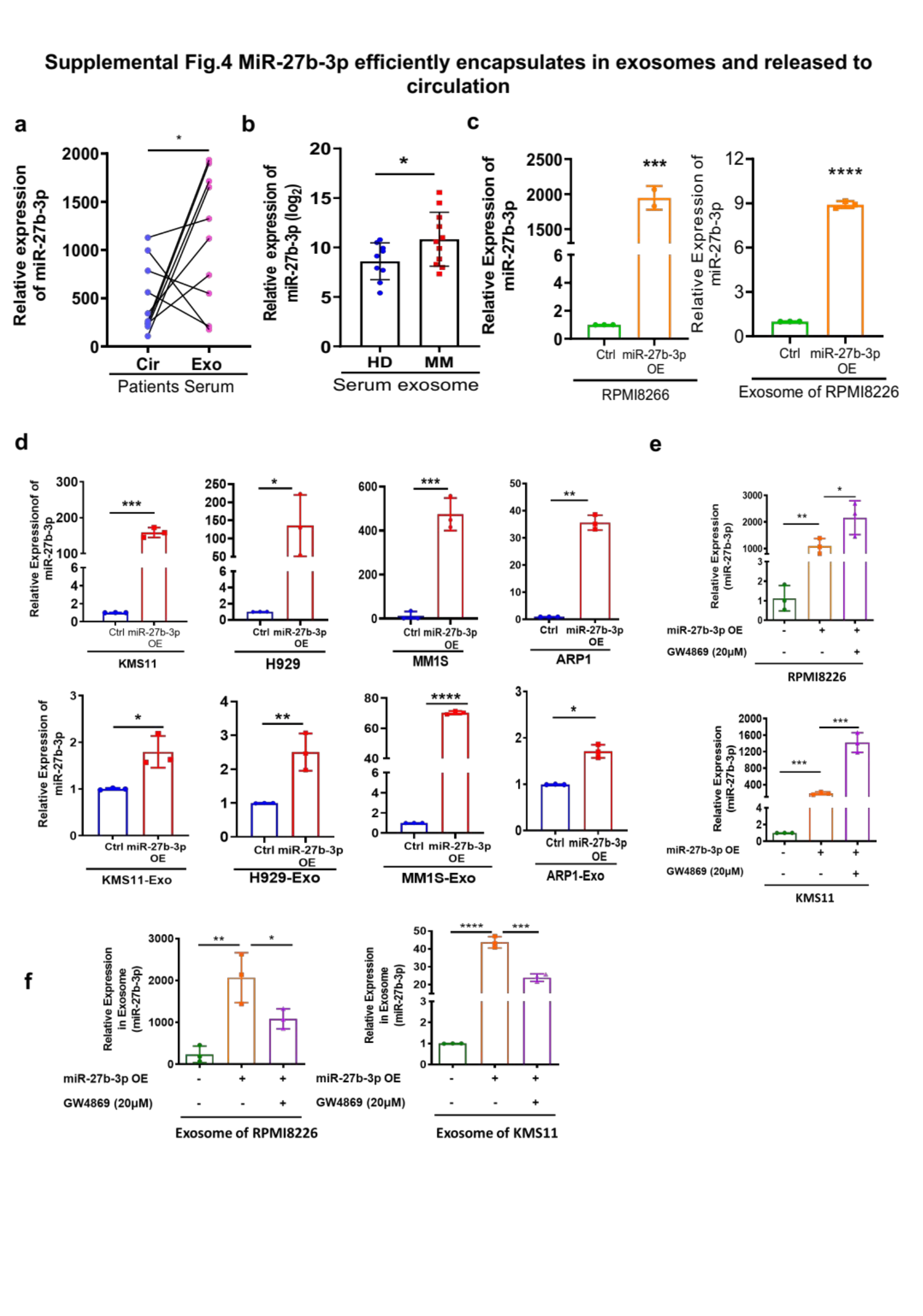

## Slide 5
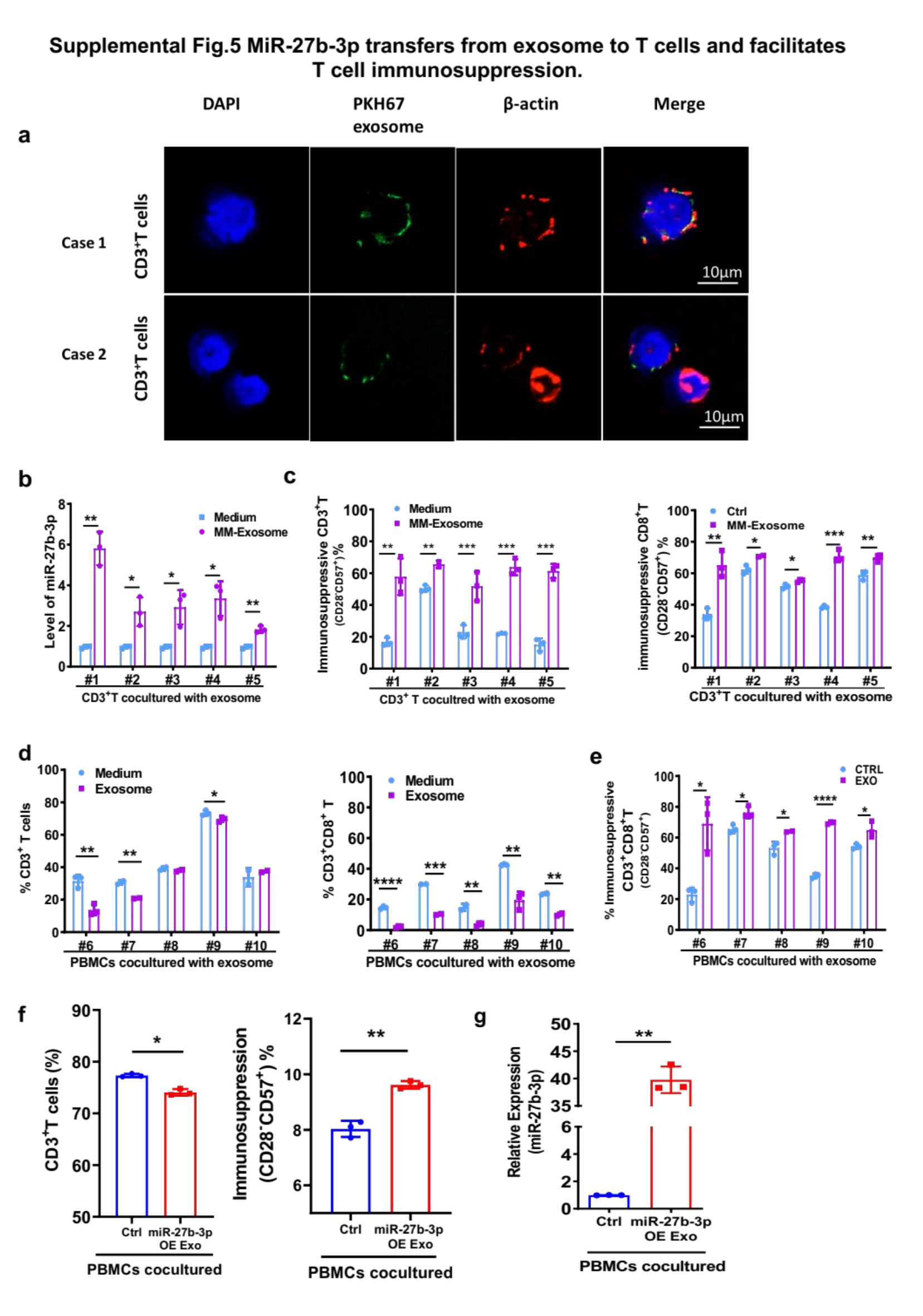

## Slide 6
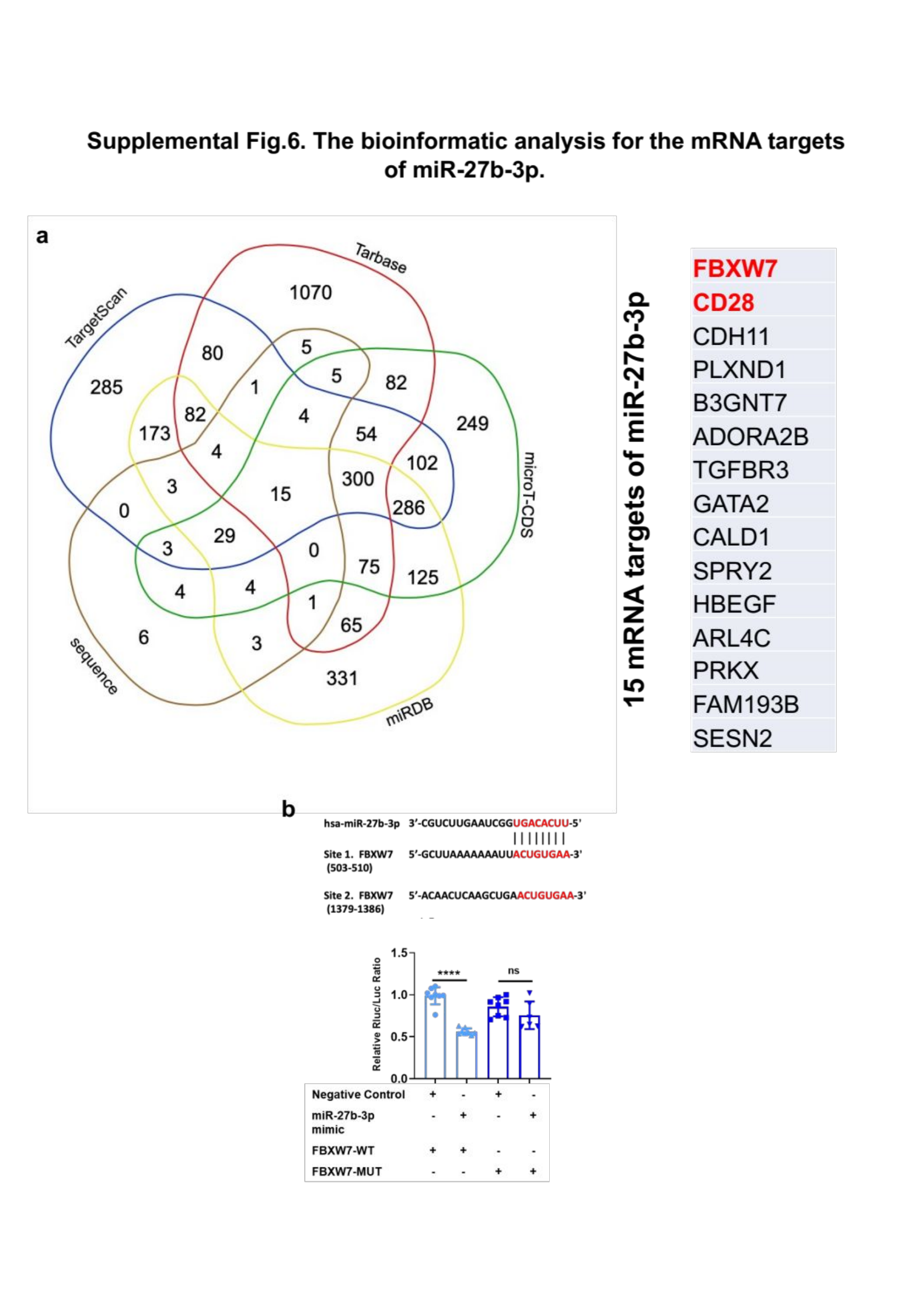

## Slide 7
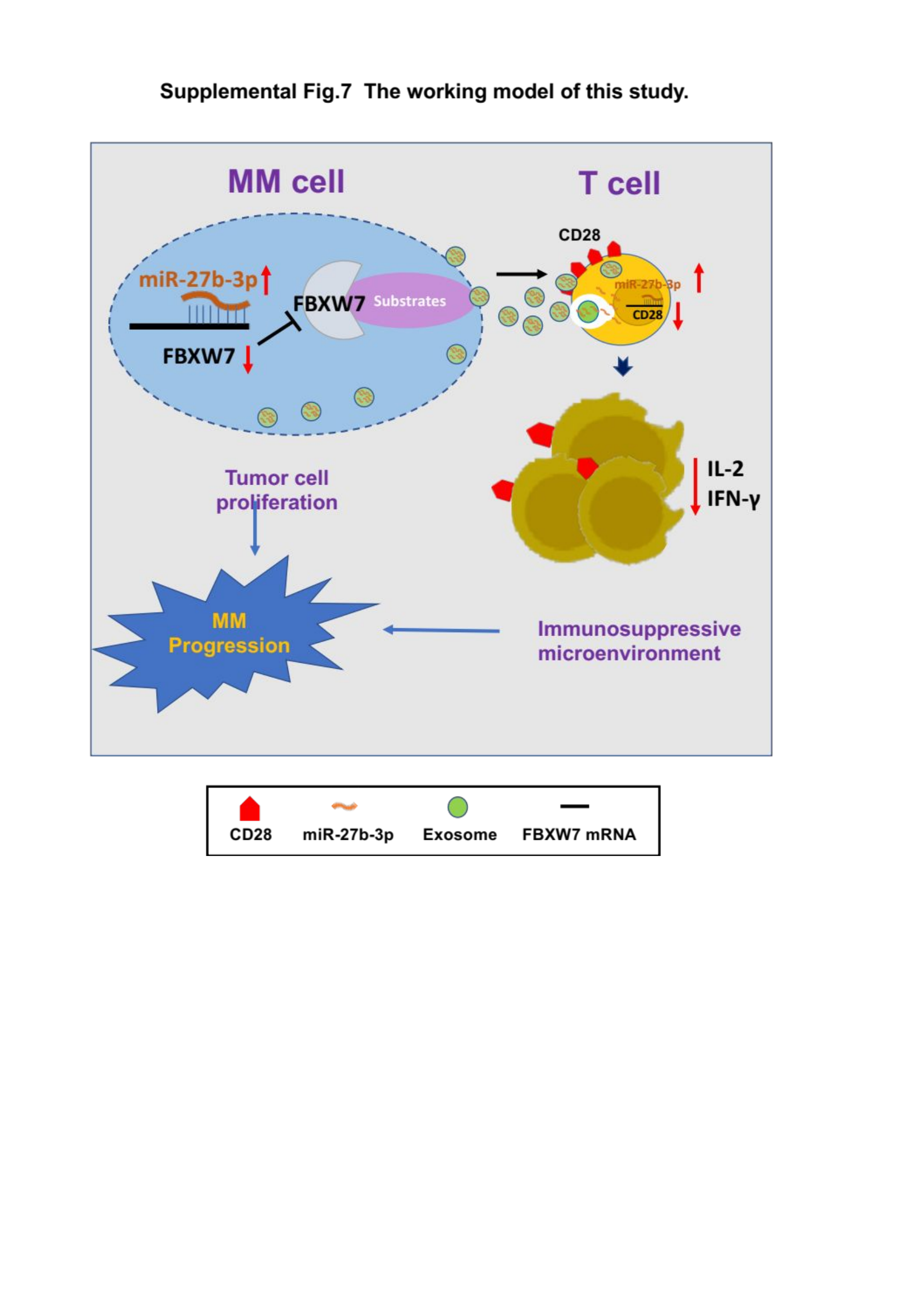

Supplement: Supplementary file 2 — Figures S1–S7 [file CTM2-13-e1140-s002.pptx]
